# Hemoglobin Targets for Chronic Kidney Disease Patients with Anemia: A Systematic Review and Meta-analysis

**DOI:** 10.1371/journal.pone.0043655

**Published:** 2012-08-30

**Authors:** Zhou Jing, Yuan Wei-jie, Zhu Nan, Zhou Yi, Wang Ling

**Affiliations:** Department of Nephrology The First People's Hospital, Shanghai Jiaotong University, Shanghai, People's Republic of China; University of Florida, United States of America

## Abstract

**Background:**

Numerous studies have identified a relationship between hemoglobin (Hb) levels and mortality in patients with chronic kidney disease (CKD), which have raised concerns about the optimal Hb targets in correction of anemia. Our study is designed to investigate the potential effects of targeted Hb levels, aiming to give some evidence for therapy of renal anemia.

**Methodology/Principal Findings:**

A comprehensive search of Medline, Embase and the Cochrane Database of Systematic Reviews was performed in December 2011 and updated in February 2012 for any new trials. Randomized trials designed to evaluate effects of high (generally the Hb about 13.0 g/dL) and low Hb (generally the Hb about 10.0 g/dL) targets on clinical outcomes in CKD patients with anemia were collected. All statistical analysis was calculated using the RevMan software available free from the Cochrane Collaboration. 24 trials involving 10361 patients were identified. Our findings demonstrated a statistically significant increased risk of mortality in the high Hb levels (RR 1.18; 95% CI 1.02 to 1.37) while the high and low Hb groups were both treated with ESAs. Overall, compared with low Hb levels, high Hb levels are associated with increased risk of hypertension (RR 1.40; 95% CI 1.11 to 1.75), stroke (RR 1.73; 95% CI 1.31 to 2.29), and hospitalizations (RR 1.07; 95% CI 1.01 to 1.14). However, there are no significant differences in the risk of non-fatal myocardial infarction (RR 1. 13; 95% CI 0.79 to 1.61) and renal replacement therapy (RR 1. 00; 95% CI 0.85 to 1.18).

**Conclusions/Significances:**

Targeting low Hb levels are beneficial to CKD patients especially in the predialysis population. The optimal Hb targets to aim for in CKD patients and at what Hb level the risks of adverse events begin to increase remain elusive. Future studies are still needed to elucidate these questions.

## Introduction

Anemia, as an inevitable and frequent complication of chronic kidney disease (CKD), is often accompanied by a wide range of clinical symptoms, such as impaired physical capacity, decreased neurocognitive function and poor quality of life both in nondialysis and dialysis patients [1]. Recently, it has been appreciated that anemia begins to develop early in the course of CKD, and the prevalence of anemia in stage 3–5 CKD was 12.0% [2]. Anemia also is an established risk factor for adverse cardiovascular outcomes, and decrease of hemoglobin (Hb) levels is highly associated with reduced production of erythropoietin (EPO). Thus, Erythropoiesis-stimulating agents (ESAs) are always considered to be an alternative therapy and being extensively used for correction of anemia [3]. However, the optimal Hb concentration to be obtained with ESAs remains a matter demanding intense discussion and getting more and more attention following publication of two large randomized trials (CHOIR and CREATE) [4], [5].

The National Kidney Foundation-Kidney Disease Outcomes Quality Initiative (NKF-KDOQI) guidelines (2000) recommended that the selected Hb targets should generally be maintained in the range of 11.0 to 12.0 g/dL in patients with CKD, whether or not they were receiving dialysis [6]. The updated KDOQI guidelines in 2006 proposed the Hb level could be expanded to the target range of 11.0 to 13.0 g/dL, with an increase in the upper limit on basis of a finding that high Hb targets mean potential improvement in patients' quality of life [7], [8]. The 2007 KDOQI guidelines indicated targeting Hb levels should not exceed 13.0 g/dL [9]. Recent guidelines for clinical practice (2009—2012) suggested that Hb targets should be in the range of 10.0—12.0 g/dL [10]. Accordingly, it is unsurprising that Hb targets have become a matter of considerable interest and consistently being explored by numerous clinical trials. Our study was set up to investigate by meta-analysis if high Hb levels associate in CKD patients significantly more outcome risk factors than low Hb levels.

## Methods

### Literature Search

A systematic literature search of Medline, Embase and the Cochrane Database of Systematic Reviews was performed in December 2011 and updated in February 2012 for any new trials, using “chronic kidney disease”, “anemia” and “erythropoiesis-stimulating agents” or “Hematinics” as the search terms. Table S1 includes the full search strategies. Only randomized controlled trials (RCTs) were included in our study. And no language limitation was applied during the retrieval process. Title and abstract of each article identified in the initial step of retrieval was screened carefully for inclusion. All potentially relevant studies were then examined in full text for further consideration. Reviews and meta-analyses regarding the optimal Hb targets in correction of renal anemia were also checked for any potential trials. As well, the cited references of all included studies were scanned to identify additional trials or reviews. Furthermore, trials were analyzed as a whole using all related reports while it had more than one publication.

### Selection Criteria

Randomized, controlled trials (RCTs) that were of at least 6 months follow-up and designed to test the efficacy and safety of Hb levels on clinical outcomes in non-dialysis and dialysis CKD patients were identified. The targeted high and low Hb levels could be achieved by different doses of ESAs, or ESAs and other feasible treatment such as placebo or no treatment at investigator's discretion. Adult patients (over 18 years old) were eligible for inclusion criteria if they had chronic anemia related to CKD. For the initial level of screening process, the main exclusion criteria were as follows: surrogate and hemorheological outcomes, pharmacokinetics and basic studies, repeated analysis and reviews or editorials.

### Validity assessment

Methodological quality and risk of bias of each included RCT were examined carefully using the approach described by the Cochrane Collaboration [11]. The items were as follows. (1) random sequence generation, (2) allocation concealment, (3) blinding of participants and personnel, (4) blinding of outcome assessment, (5) incomplete outcome data, (6) selective reporting and (7) other sources of bias. All seven items were classified as “low risk of bias”, “high risk of bias”, or “unclear risk of bias”. Moreover, the intention-to-treat analysis principle (ITT) was also used to evaluate the integrity of those outcome data, and the grade tool was used to grade the quality of evidence and the strengths of recommendations.

### Data Extraction

Data were extracted independently from each RCT that fulfilled the inclusion criteria, and any discrepancies were figured out by consensus. These obtained data were then examined by another reviewer for its accuracy. Attempts to contact the trial authors were also made for additional information when the outcomes were not reported or the data was not comprehensive. Data were extracted from the included trials based on a standardized strategies, including details of the publication, design principle of the trial, size of study population, characteristics of participants (such as CKD stage, age, gender, sex and dialysis modality), interventions (such as the dosages of ESAs and iron treatment), primary end points (such as mortality, non-fatal myocardial infarction, stroke, hypertension, hospitalizations and renal replacement therapy), the follow-up duration and the Hb values (including the baseline Hb levels, the targeted Hb levels and the achieved Hb levels).

### Quantitative data synthesis

We grouped studies according to mode of treatment as described above into the high Hb versus low Hb group and ESAs treatment versus no ESAs treatment group. Results were also calculated separately as subgroup analysis.

Data from each included trials were analyzed using Review Manager (RevMan, Version 5.1, Copenhagen: The Nordic Cochrane Centre, The Cochrane Collaboration, 2011). Results were presented as relative risk (RR) and its 95% confidence intervals (CI). Statistical heterogeneity within and between studies was described using the χ^2^ test, and the *I* ^2^ test for inconsistency. The degree of inconsistency across studies was defined by calculating the percentage of total between-study variation because of heterogeneity rather than random variation as the *I* ^2^ metric using the formula *I* ^2^ = 100%×(Q-df)/Q [12]. For dichotomous data, we calculated the Mantel-Hansel risk ratio using a random-effects model when heterogeneity was present with a significant result of p<0.1. Mild, moderate, and severe heterogeneity was defined by I^2^ values at >25%, >50%, and >75%, respectively. As for the dichotomous outcomes, the DerSimonian and Laird random-effects model was used when heterogeneity across the trials was significant [13].

A funnel plot was used to assess the presence of publication and other reporting biases by plotting the standard error against the log risk ratio. Using Egger's linear regression method, we examined the association between the study size and estimated treatment effects. P≤0.05 was considered significant. The P-value threshold for statistical significance was set at 0.05 for effect size.

## Results

### Trial Flow/Flow of included studies

**Figure 1:**
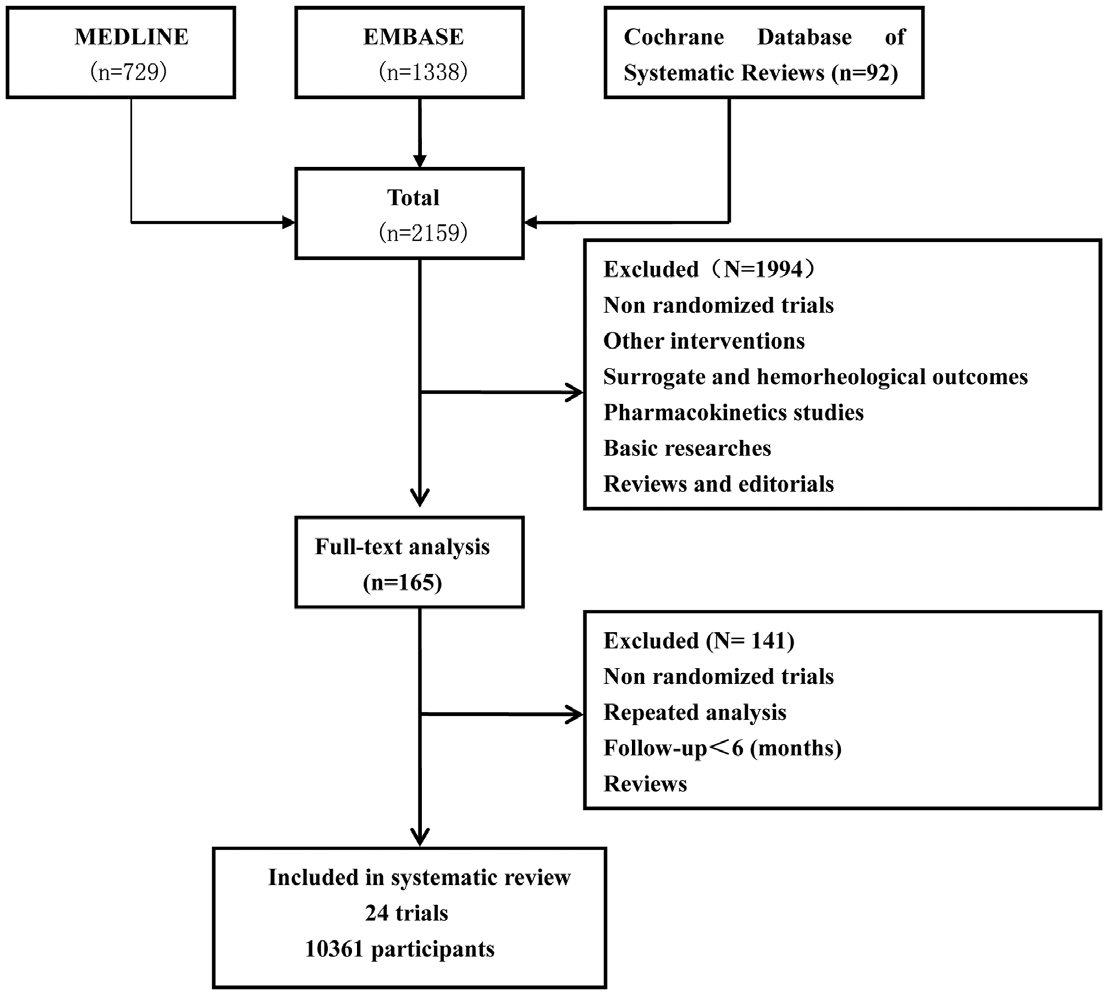
Flowchart demonstrating the literature screening process and reasons for exclusion are explained.

2159 trials were identified by searching Medline, Embase and the Cochrane Database of Systematic Reviews, 1994 of which were excluded in the initial screening. 165 potentially relevant trials were identified for further review, of which 24 trials fulfilled our inclusion criteria. All selected studies were designed as randomized controlled trials enrolling patients with CKD anemia (Figure 1).

### Study characteristics

24 randomized trials (10361 participants) designed to evaluate efficacy and safety of Hb levels on clinical outcomes in patients with renal anemia were collected. The baseline characteristics of the 24 trials are listed in table 1 and table S2, S3 [4], [5], [14]–[35]. Most of the participants were randomized to achieve two pre-established Hb targets. Based on the treatment modality, we divided these studies into two subgroups: the high and low Hb levels in 15 trials were obtained with ESAs therapy, and in the rest 9 trials, the high Hb group were treated with ESAs while the low Hb group were given no treatment or treated with placebo. Generally, the high Hb targets were achieved with a higher dose of EPO while the low Hb targets were achieved with a lower dose.

**Table 1:** Baseline Characteristics of the Participants.

Study	CKD Stage	Age (H^a^)	Age (L^b^)	Gender(%) (F/M) (H^a^)	Gender(%) (F/M)(L^b^)	Diabetes (%)H^a^(L^b^)	Hypertension (%)H^a^(L^b^)
High Hb target versus Low Hb Target							
Singh et al. [4]	3–4	66.0±14.3	66.3±13.5	56.2/43.8	54.1/45.9	NA^d^	95.8(93.2)
Drueke et al. [5]	3–4	59.3±14.6	58.8±13.7	43/57	49/51	27(25)	91(89)
Akizawa et al. [14]	3–4	65.2±11.8	64.1±11.7	50.3/49.7	55.6/44.4	NA^d^	83.9 (83.8)
Villar et al. [15]	3–4	68.5±7.6	65.2±9.1	39.1/60.9	34.9/65.1	100(100)	97.8 (100)
Cianciaruso et al. [16]	2–5	58.5±14.6	56.5±14.4	43.5/56.5	32.7/67.3	23.9(12.2)	NA^d^
Ritz et al. [17]	1–3	49–69	47–66	49/51	50/50	100(100)	0(0)
Parfrey et al. [18]	5	52.2±15.6	49.2±15.4	40/60	40/60	19(17)	7(9)
Levin et al. [19]	3–4	56.5±14.9	57.3±14.9	45/55	48/52	41(35.1)	NA^d^
Roger et al. [20]	3–4	53±14(M),50±14(F)	54±12(M),50±15(F)	49/51	58/42	24 (33)	74(74)
Gouva et al. [21]	3–5	66.7±10.2	64.2±12.2	44.4/53.6	41.9/58.1	NA^d^	93(84)
Furuland et al. [22]	5	63±12	63±14	33/67	37/63	19(20)	52(48)
Foley et al. [23]	5	57–67	56–65	56/44	53/47	NA^d^	NA^d^
Conlon et al.^c^ [24]	5	53.1±12	56.2±13	63/37	47/53	NA^d^	NA^d^
Berns et al.^c^ [25]	5	63.8±12.7	58.6±8.9	71.4/29.6	64.3/35.7	50(57.1)	NA^d^
Besarab et al. [26]	5	65±12	64±12	50/50	52/48	54(58)	71(69)
CESG(a substudy) [27]	5	43±15	44±16	39.5/60.5	52.5/47.5	NA^d^	NA^d^
ESA treatment versus no ESA treatment							
Patel et al. [28]	1–4	84.1±9.2	84.4±10.9	76.3/23.7	84.6/15.4	NA^d^	NA^d^
Preffer et al. [29]	2–4	68	68	58.5/41.5	56.0/44	100(100)	NA^d^
Pappas et al. [30]	3–4	64.4±11.8	66.2±10.5	53.3/46.7	37.5/63.5	NA^d^	NA^d^
Kuriyama et al. [31]	3–5	63.8±10.6	59.2±13.4	45/55	48/52	55(58)	78(76)
Revicki et al. [32]	4–5	56.5±11.4	58.4±13.2	70/30	65/35	NA^d^	NA^d^
Nissenson et al. [33]	5	46.8±15.5	49.9±15.9	61/39	48/52	27(24)	86(92)
Sikole et al. [34]	5	25–70	23–69	42.1/57.9	47.4/52.6	NA^d^	NA^d^
Teehan et al. [35]	5	53.8±10.6	56.8±10.9	59/41	57.5/42.5	NA^d^	NA^d^
CESG [27]	5	43±15	48±16	31.6/68.4	53.5/46.5	NA^d^	NA^d^

a **H, High Hb Group.**

b **L, Low Hb Group.**

c **Participants have severe cardiac disease, eg. Congestive heart disease or ischemic cardiac disease.**

d **NA, not available.**

Overall, the mean achieved Hb levels in the 15 trials were 13.0 g/dL in the high Hb group and 10.0 g/dL in the low Hb group. As for the rest 9 trials, patients who were randomized to ESAs treatment group ended up with achieved Hb level about 12.0 g/dL, while those randomized to placebo or no treatment group having Hb level around 9.0 g/dL. In our included studies, participants were generally older than 18 years. Approximately 75% of those participants had CKD stage III–V. Furthermore, the Villar et al. [15] and Pfeffer et al. [29] enrolled patients with type 2 diabetes mellitus, and the Ritz et al. [17] enrolled patients with type 1 or type 2 diabetes mellitus. The Conlon et al. [24], Berns et al. [25] and Besarab et al. [26] enrolled patients with severe cardiac disease (such as congestive heart failure and ischemic heart disease). Most of them have mild-to-moderate anemia, and patients in the Gouva et al. [21] and Kuriyama et al. [31] have moderate-to-severe anemia.

Generally the same ESAs products were given to patients for therapy of anemia. In the Akizawai et al. [14] trial, patients were treated with different kinds of ESAs products in the two groups, darbepoetin alfa for the high Hb arm and rHuEPO for low Hb arm. Meanwhile, Gouva et al. [21] and Pappas et al. [30] used an alternative analogue of rhuEPO (e.g. rhuEPO beta or darbepoietin) to substitute for rhuEPO-alfa due to reports of pure red-cell aplasia cases (PRCA).

### Quantitative data synthesis

**Figure 2:**
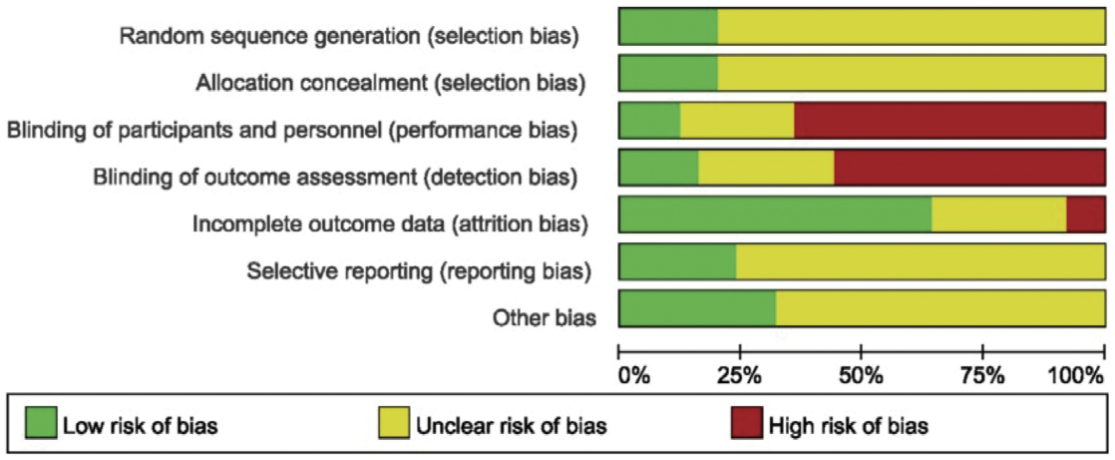
Risk of bias graph available according to recommendations from the Cochrane Collaboration.

According to the standard methods recommended by the Cochrane Collaboration for assessing risk of bias [11], trial quality was not optimal (Figure 2). Detail of allocation concealment only in one trial was clearly clarified (Table 2), and only 14 trials of those included studies were analyzed based on the intent-to-treat analysis (ITT) principle. The quality of evidence and the strengths of recommendations were listed in table S4.

**Table 2:** Details on risk of bias assessment.

Study	Blinding of participants	Blinding of outcome	Generation of allocation	Allocation
	and personnel	assessment		concealment
Singh et al. [4]	No	Unclear	Computer-generated permuted	Unclear
			block randomization	
Drueke et al. [5]	No	Unclear	Unclear	Unclear
Akizawa et al. [14]	No	No	By computer	Unclear
Villar et al. [15]	No	No	By a block-size	Unclear
			randomization procedure	
Cianciaruso et al.	No	No	Computer generated	Yes*
[16]			randomization lists	
Ritz et al. [17]	No	No	By a block-size	Unclear
			randomization procedure	
Parfrey et al. [18]	Yes	Unclear	by an interactive voice randomization	Unclear
			telephone system, using permuted	
			blocks	
Levin et al. [19]	No	Yes	randomization lists generated	Unclear
			by a permuted variable block design	
Roger et al. [20]	No	No	randomization codes generated	Unclear
			by telephoning a central number	
Gouva et al. [21]	Unclear	Unclear	by a computerized sequence	Unclear
Furuland et al. [22]	No	No	Unclear	Unclear
Foley et al. [23]	No	No	Unclear	Unclear
Conlon et al. [24]	Unclear	Unclear	Unclear	Unclear
Berns et al. [25]	No	Unclear	Unclear	Unclear
Besarab et al. [26]	No	Unclear	Unclear	Unclear
CESG (a substudy)	Yes	Unclear	By a block-size	Unclear
[27]			randomization procedure	
CESG [27]	Yes	Unclear	By a block-size	Unclear
			randomization procedure	
Patel et al. [28]	Yes	No	Unclear	Unclear
Pfeffer et al. [29]	Yes	No	A computer-generated,	Unclear
			permuted-block design	
Pappas et al. [30]	Unclear	Unclear	Unclear	Unclear
Kuriyama et al. [31]	Unclear	Unclear	Unclear	Unclear
Revicki et al. [32]	No	Unclear	Unclear	Unclear
Nissenson et al. [33]	Yes	Unclear	By a block-size	Unclear
			randomization procedure	
Sikole et al. [34]	Unclear	Unclear	Unclear	Unclear
Teehan et al. [35]	Unclear	Unclear	Unclear	Unclear

* sealed opaque envelops.

#### Mortality

**Figure 3:**
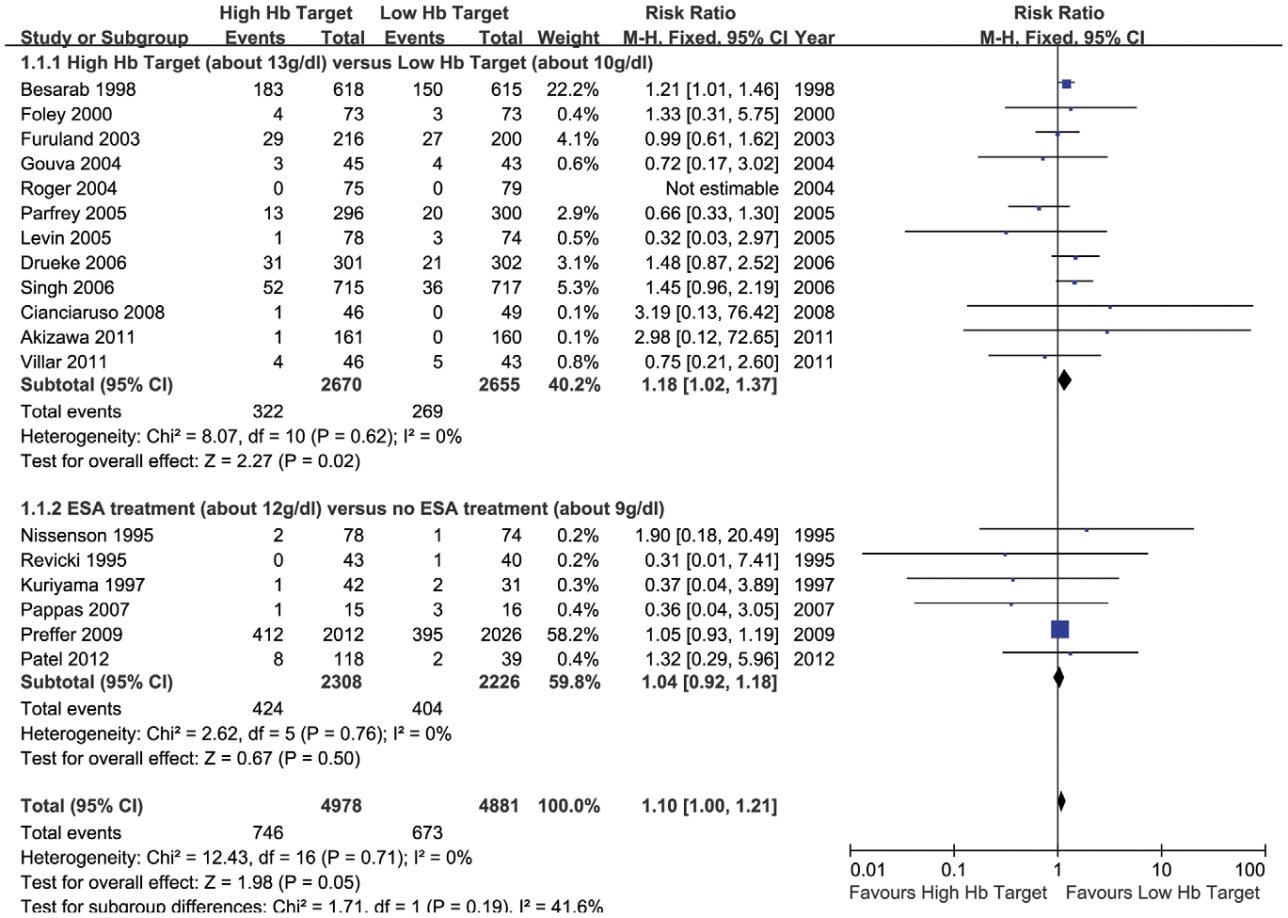
Meta-analysis of high Hb targets versus low Hb targets on mortality.

**Figure 4:**
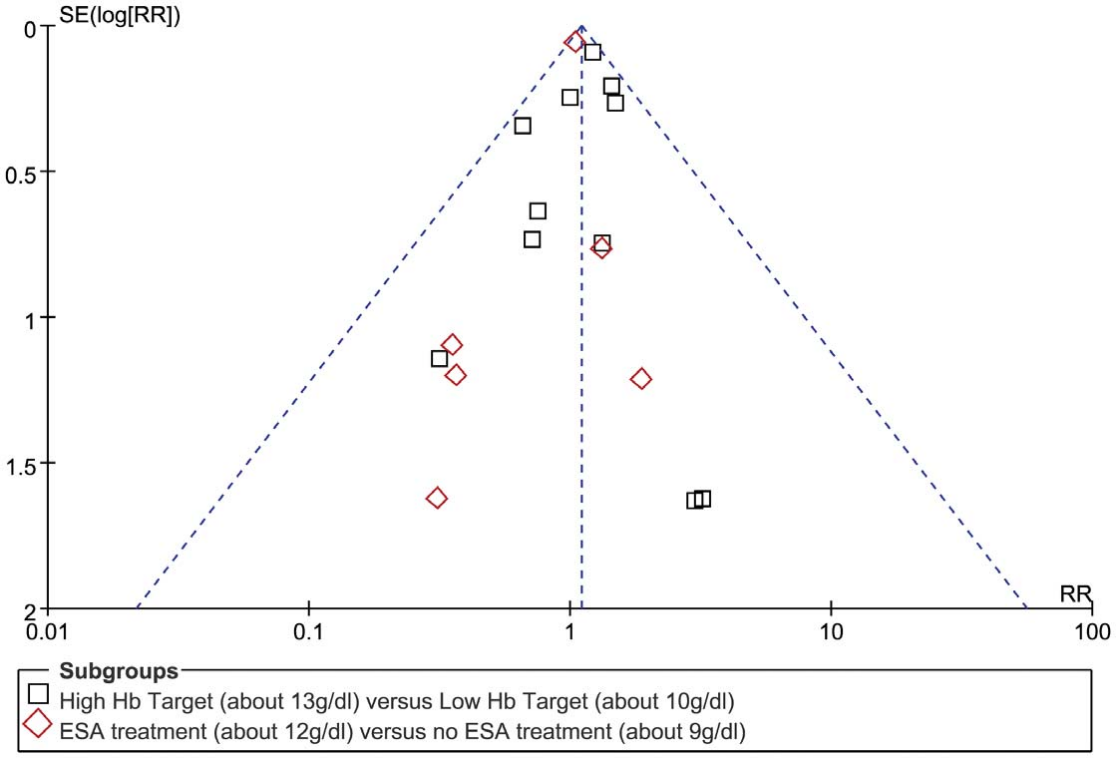
Funnel plot with 95% CI to evaluate publication bias in the two subgroups. One study with no events in both groups was excluded.

In the high Hb target versus low Hb target trials, a borderline significance was detected in the risk of mortality with the high Hb group compared with the low Hb group (18 trials, 9859 patients; RR 1.10; 95% CI 1.00 to 1.21; P = 0.05). This analysis was dominated by the Pfeffer et al. [29] study, contributing 58.2% of the weight. Heterogeneity across these trials was also not significant (heterogeneity X^2^ = 12.43, P = 0.71, I^2^ = 0%) (Figure 3).The funnel-plot analysis did not show asymmetry demonstrating that the publication bias cannot substantially affect the result of this meta-analysis (Figure 4).

In the ESAs treatment versus no ESAs treatment group, a fix-effect model was applied as a result of no heterogeneity across the trials (heterogeneity X^2^ = 2.62, P = 0.76, I^2^ = 0%). No statistically significant difference was detected in the risk of all-cause mortality between the two groups (6 trials, 4534 patients; RR 1.04; 95% CI 0.92 to 1.18; P = 0.50). In the trials of both groups treated with ESAs, there was a higher risk of death with the high Hb group compared with the low Hb group and it has a significant statistical significance (12 trials, 5325 patients; RR 1.18; 95% CI 1.02 to 1.37; P = 0.02) even though some of the studies (the Villar et al. [15], Parfrey et al. [18], Levin et al. [19], Gouva et al. [21] and Furuland et al. [22]) show an opposite direction of effects, and the Besarab et al. [26] study accounts for a large proportion of this analysis. There was no heterogeneity across these trials (heterogeneity X^2^ = 8.07, P = 0.62, I^2^ = 0%).

#### Hypertension

A total of 9 trials were identified to evaluate occurrence of hypertension in different Hb targets. There was a higher risk of hypertension in the high Hb target as compared with the low Hb target with statistical significance (9 trials, 6756 patients; RR 1.40; 95% CI 1.11 to 1.75; P = 0.004). Heterogeneity was demonstrated significantly in the group of studies (heterogeneity X^2^ = 18.55, P = 0.02, I^2^ = 57.0%).

3 trials (including a substudy of the Canadian Eryhtropoietin Study Group trial) were identified as ESAs treatment versus no ESAs treatment. There was a statistically significant higher risk of hypertension in the ESAs treatment arm compared with no ESAs treatment arm (3 trials, 4199 patients; RR 1.13; 95% CI 1.01 to 1.26; P = 0.04). Heterogeneity in this group of studies was not significant (heterogeneity X^2^ = 3.39, P = 0.18, I^2^ = 41.0%).

6 studies in trials of both groups treated with ESAs provide data on the number of patients having hypertension. A significantly increased risk of hypertension was detected in the high Hb level as compared with low Hb level with statistical significance (6 trials, 2557 patients; RR 1.49; 95% CI 1.08 to 2.05; P = 0.01). and heterogeneity is significant across these trials (heterogeneity X^2^ = 12.35, P = 0.03, I^2^ = 60.0%) (Figure S1).

#### Stroke

This outcome was reported in 4 trials, one of which in the ESAs treatment versus no ESAs treatment arm while 3 in the both ESAs treated group. There was a higher risk of stroke in the ESAs treatment group compared with group with no ESAs treatment with significant statistical significance (1 trial, 4038 patients; RR 1.92; 95% CI 1.38 to 2.66; P<0.0001).

As for the groups both treated with ESAs, no statistically significant difference in the risk of stroke was detected between the two groups (3 trials, 2754 patients; RR 1.27; 95% CI 0.73 to 2.23; P = 0.40). Heterogeneity in this group of studies was not significant (heterogeneity X^2^ = 0.80, P = 0.67, I^2^ = 0%).

In the high Hb target versus low Hb target trials, there was a statistically significant higher risk of stoke in the high Hb target compared with low Hb target (4 trials, 6792 patients; RR 1.73; 95% CI 1.31 to 2.29; P = 0.0001). Heterogeneity in this group of studies was not significant (heterogeneity X^2^ = 2.36, P = 0.50, I^2^ = 0%). And this study was dominated by the Pfefffer et al. trial, contributing 71.0% of the weight (Figure S2).

#### Non-fatal Myocardial Infarction

This outcome was reported only in the trials of both groups treated with ESAs. There was no statistically significant difference in risk of non-fatal myocardial infarction between the two arms (6 trials, 4123 patients; RR 1.13; 95% CI 0.79 to 1.61; P = 0.51). Heterogeneity in this group of trials was not significant (heterogeneity X^2^ = 2.41, P = 0.79, I^2^ = 0%) (Figure S3).

#### Renal Replacement Therapy

12 trials were included in this section, 3 of these were from the group of studies comparing ESAs treatment versus no ESAs treatment, and 9 were from studies both arms treated with ESAs. No statistically significant difference in the risk of renal replacement therapy was demonstrated both in the ESAs treatment versus no ESAs treatment (3 trials, 4194 patients; RR 0.86; 95% CI 0.56 to 1.34; P = 0.52) and the high and low Hb arms both treated with ESAs (9 trials, 3104 patients; RR 1.10; 95% CI 0.97 to 1.25; P = 0.15). Heterogeneity was significant in the group of ESAs treatment versus no ESAs treatment (heterogeneity X^2^ = 7.08, P = 0.03, I^2^ = 72.0%), while no heterogeneity was detected in the both ESAs treated group (heterogeneity X^2^ = 11.15, P = 0.19, I^2^ = 28.0%).

To summary, there was no statistically significant difference in the risk of renal replacement therapy between the high Hb target and low Hb target (12 trials, 7298 patients; RR 1.00; 95% CI 0.85 to 1.18; P = 1.00). Heterogeneity in this group of studies was significant (heterogeneity X^2^ = 19.65, P = 0.05, I^2^ = 44.0%) (Figure S4).

#### Hospitalizations

This outcome was analyzed only in the trials of both groups treated with ESAs. There was a statistically significant higher risk of non-elective hospitalizations in the high Hb group compared with low Hb group (3 trials, 2756 patients; RR 1.07; 95% CI 1.01 to 1.14; P = 0.03). This analysis was dominated by the Besarab et al. [26] study which contributes 55.6% of the weight while the Gouva et al. [21] contributing 0.8% of the weight demonstrated an opposite direction of effects. Heterogeneity in this group of trials was demonstrated (heterogeneity X^2^ = 1.49, P = 0.48, I^2^ = 0%) (Figure S5).

## Discussion

We observed an increased risk of mortality in patients having high Hb levels, as compared with patients having low Hb levels when both groups were treated with ESAs, though no significant difference was detected in the groups of ESAs treatment versus no ESAs treatment. Only a borderline difference was found in mortality favouring lower Hb concentrations if we just take the high and low Hb levels into account irrespective of treatment modality. Recently, many reviews and meta-analyses exploring the optimal Hb targets for CKD patients with anemia have been published. Our results are consistent with the findings of a systematic review by Strippoli et al. [36], which indicated maintaining low Hb levels in CKD patients is associated with decreased risk of hypertension and death when compared with the high Hb levels. Another meta-analysis of 9 RCTs involving 5143 participants also demonstrated that normalizing Hb was associated with increased risk of mortality and uncontrolled blood pressure [37], which is in consistent with our meta-analysis that includes the most recently published clinical trials. However, the most recently published is a “meta-analysis” of ESAs in patients with CKD [38], which found no statistically significant difference in the risk of mortality between the high and low Hb levels. Considering therapy of anemia with erythropoietin may induce or worsen the occurrence of hypertension, and the Trial to Reduce Cardiovascular Events with Aranesp Therapy (TREAT) conducted in predialysis patients with CKD stages 2, 3 or 4 and diabetes found no reduced risk of death in the ESAs treated group compared with placebo-controlled group, and treating anemia with ESAs maybe harmful [29], We divided the high and low Hb targets into another two subgroups based on whether or not the patients were given ESAs for anemia treatment, and the statistical analysis was also computed separately. As a result, our findings as described above are less influenced by the side-effects of ESAs itself. Furthermore, our present finding is applicable mainly to the predialysis patients. Recently, two large randomized, controlled trials of the Correction of Hemoglobin and Outcomes in Renal Insuffiency (CHOIR) conducted in predialysis patients with CKD stages 3 or 4 found that targeting a high Hb level (13.5 g/dl) was associated with increased risk of composite events (including death and stroke) compared with low Hb target (11.3 g/dl), and the Cardiovascular Risk Reduction by Early Anemia Treatment with Epoetin Beta (CREATE) conducted in predialysis patients with CKD stages 3 or 4 conformed that early and complete correction of anemia does not reduce the risk of cardiovascular events which could contribute to the increased risk of mortality [4], [5].

As for the dialysis patients, there was no statistically significant difference in the risk of mortality between the two Hb levels. However, an epidemiogical study revealed high hematocrit levels were favourable to dialysis patients of different age, gender and race [39]. McMahon et al. [40] found that maintaining high Hb concentrations have a significant hemodynamic and symptomatic advantage in haemodialysis patients, as compared with low Hb concentrations. Despite this, there is still a possibility that high Hb targets are associated with increased risk of mortality in dialysis patients. Reportedly, Parfrey et al. trial [18] was prematurely terminated mainly as a result of a trend toward greater mortality in the high-haematocrit group compared with low-haematocrit group. Furthermore, another large, randomized and controlled trial by Besarab et al. [26] was halted early when unexpected excess risk of death was detected in the near-normal target arm, and continuation of this study seems unlikely to reveal a benefit for the higher Hb group. Hence, these trials, to some extent, helped to confirm the finding that high Hb targets is associated with increased risk of mortality in dialysis patients.

In our review, high Hb levels also is associated with a significantly increased risk of hypertension when compared with low Hb levels, and this appears to be consistent and independent of whether patients were treated with ESAs or not. Low Hb targets are associated with reduced risk of stroke compared with high Hb targets (RR 1.73; 95%CI 1.31 to 2.29; P = 0.0001). Finally, high Hb levels are associated with an increased risk of hospitalizations compared with low Hb levels (RR 1.07; 95% CI 1.00 to 1.14; P = 0.04).

Altogether, our results may shed some light on the link among greater risk of mortality, hypertension, stroke, hospitalizations and high Hb targets in correction of anemia in CKD patients. Overall, results from this meta-analysis strongly suggests that for patients with anemia associated with chronic kidney disease, maintaining high Hb targets in all CKD patients, including those with diabetes and cardiovascular disease, may be unfavourable. Nowadays, many nephrologists proposed that partial correction of anemia to maintain Hb levels in the target range of 10–12 g/dL was a safe strategy. Nonetheless, recently released modified recommendations for usage of ESAs in patients with CKD suggest lowering the ESAs dose while Hb levels is more than 10 g/dL and 11 g/dL, respectively, in predialysis and dialysis patients. And the previously recommended Hb targets around 10–12 g/dL had been removed from its label [41]. It is likely that the recent FDA labeling update would increase the risk of blood transfusions and lower quality of life. Hence, the appropriate Hb levels to start ESAs treatment, the optimal Hb targets to aim for in CKD patients and at what Hb level the risk of adverse events begin to increase remain elusive. Future studies of longer duration in dialysis and predialysis patients focusing on the association between clinical outcomes and Hb levels or/and ESAs treatment are still needed. Accordingly, clinicians should balance risks and benefits of each patient when using ESAs for anemia management and individualized therapy for each patient may also be advocated.

## Limitations

Certain limitations in our study need to be considered. (1) Several studies eligible for the inclusion criteria were not powered to evaluate mortality; (2) Three of our included trials enrolled patients with cardiac disease and another three studies enrolling patients with diabetes; (3) Different kinds of ESAs were used in three trials of our study during follow-up; (4) The follow-up duration is different among those identified trials. Despite these limitations, we believe that this meta-analysis, including 24 RCTs, could indicate that maintaining low Hb targets is more favorable in therapy of renal anemia.

## Supporting Information

Figure S1 Effect of high versus low Hb targets on hypertension in patients with CKD. S1.1. High Hb versus Low Hb targets (both group treated with ESAs). S1.2. ESAs treatment versus no ESAs treatment. S1.3. Summary of the high Hb versus low Hb group (irrespective of treatment modality).
(PDF)

Figure S2 Effect of high versus low Hb targets on stroke in patients with CKD.
(PDF)

Figure S3 Effect of high versus low Hb targets on non-fatal myocardial infarction in patients with CKD.
(PDF)

Figure S4 Effect of high versus low Hb targets on RRT in patients with CKD. S4.1. High Hb versus Low Hb targets (both group treated with ESA). S4.2. ESA treatment versus no ESAs treatment. S4.3. Summary of the high Hb versus low Hb group (irrespective of treatment modality).
(PDF)

Figure S5 Effect of high versus low Hb targets on hospitalizations in patients with CKD.
(PDF)

Table S1 Search Strategy.
(PDF)

Table S2 Characteristics of studies included in our meta-analysis.
(PDF)

Table S3 Summary of Hb levels in our included studies.
(PDF)

Table S4 The quality of evidence and the strengths of recommendations.
(PDF)
