# Supplementary material for: Hemoglobin Targets for Chronic Kidney Disease Patients with Anemia: A Systematic Review and Meta-analysis
Source: PLoS One. 2012 Aug 30;7(8):e43655. doi: 10.1371/journal.pone.0043655 (PMC3431367; doi:10.1371/journal.pone.0043655)

Figure S1. **Effect of high versus low Hb targets on hypertension in patients with CKD.**

S1.1 High Hb versus Low Hb targets (both group treated with ESAs)

	High Hb target		Low Hb target			Risk Ratio
Study or Subgroup	Events	Total	Events	Total	Weight	M-H, Random, 95% CI
Akizawa 2011	17	161	11	160	12.4%	1.54 [0.74, 3.17]
Besarab 1998	116	618	112	615	28.9%	1.03 [0.82, 1.30]
CESG(a substudy)1990	2	38	2	40	2.6%	1.05 [0.16, 7.10]
Druke 2006	89	301	59	302	26.8%	1.51 [1.14, 2.02]
Nissenson 1995	39	78	14	74	17.9%	2.64 [1.57, 4.45]
Ritz 2007	15	88	9	82	11.5%	1.55 [0.72, 3.35]
**Total (95% CI)**		**1284**		**1273**	**100.0%**	**1.49 [1.08, 2.05]**
Total events	278		207			

Heterogeneity: Tau² = 0.08; Chi² = 12.35, df = 5 (P = 0.03); I² = 60%
Test for overall effect: Z = 2.45 (P = 0.01)

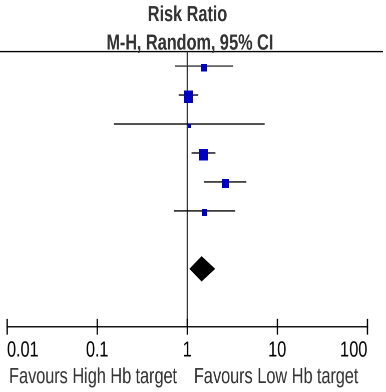

S1.2 ESAs treatment versus no ESAs treatment

	ESA treatment		no ESA treatment			Risk Ratio
Study or Subgroup	Events	Total	Events	Total	Weight	M-H, Fixed, 95% CI
CESG 1990	2	38	0	40	0.1%	5.26 [0.26, 106.06]
Pfeffer 2009	491	2012	446	2026	99.0%	1.11 [0.99, 1.24]
Revicki 1995	11	43	4	40	0.9%	2.56 [0.89, 7.39]
**Total (95% CI)**		**2093**		**2106**	**100.0%**	**1.13 [1.01, 1.26]**
Total events	504		450			

Heterogeneity: Chi² = 3.39, df = 2 (P = 0.18); I² = 41%
Test for overall effect: Z = 2.09 (P = 0.04)

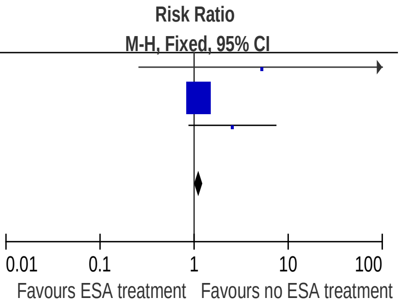

S1.3 Summary of the high Hb versus low Hb group (irrespective of treatment modality)

	High Hb target		Low Hb target			Risk Ratio
Study or Subgroup	Events	Total	Events	Total	Weight	M-H, Random, 95% CI
Akizawa 2011	17	161	11	160	7.2%	1.54 [0.74, 3.17]
Besarab 1998	116	618	112	615	22.1%	1.03 [0.82, 1.30]
CESG 1990	2	38	0	40	0.6%	5.26 [0.26, 106.06]
CESG(a substudy)1990	2	38	2	40	1.3%	1.05 [0.16, 7.10]
Druke 2006	89	301	59	302	19.7%	1.51 [1.14, 2.02]
Nissenson 1995	39	78	14	74	11.4%	2.64 [1.57, 4.45]
Pfeffer 2009	491	2012	446	2026	27.2%	1.11 [0.99, 1.24]
Revicki 1995	11	43	4	40	3.9%	2.56 [0.89, 7.39]
Ritz 2007	15	88	9	82	6.6%	1.55 [0.72, 3.35]
**Total (95% CI)**		**3377**		**3379**	**100.0%**	**1.40 [1.11, 1.75]**
Total events	782		657			

Heterogeneity: Tau² = 0.05; Chi² = 18.55, df = 8 (P = 0.02); I² = 57%
Test for overall effect: Z = 2.90 (P = 0.004)

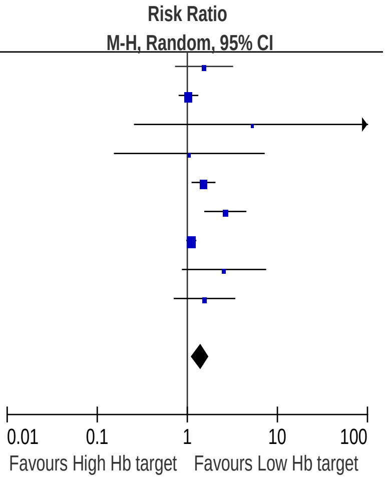

Supplement: Figure S1 — Effect of high versus low Hb targets on hypertension in patients with CKD. S1.1. High Hb versus Low Hb targets (both group treated with ESAs). S1.2. ESAs treatment versus no ESAs treatment. S1.3. Summary of the high Hb versus low Hb group (irrespective of treatment modality). (PDF) [file pone.0043655.s001.pdf]
