# Supplementary figures and images for: Hemoglobin Targets for Chronic Kidney Disease Patients with Anemia: A Systematic Review and Meta-analysis
Source: PLoS One. 2012 Aug 30;7(8):e43655. doi: 10.1371/journal.pone.0043655 (PMC3431367; doi:10.1371/journal.pone.0043655)

**Figure S2 Effect of high versus low Hb targets on stroke in patients with CKD.**

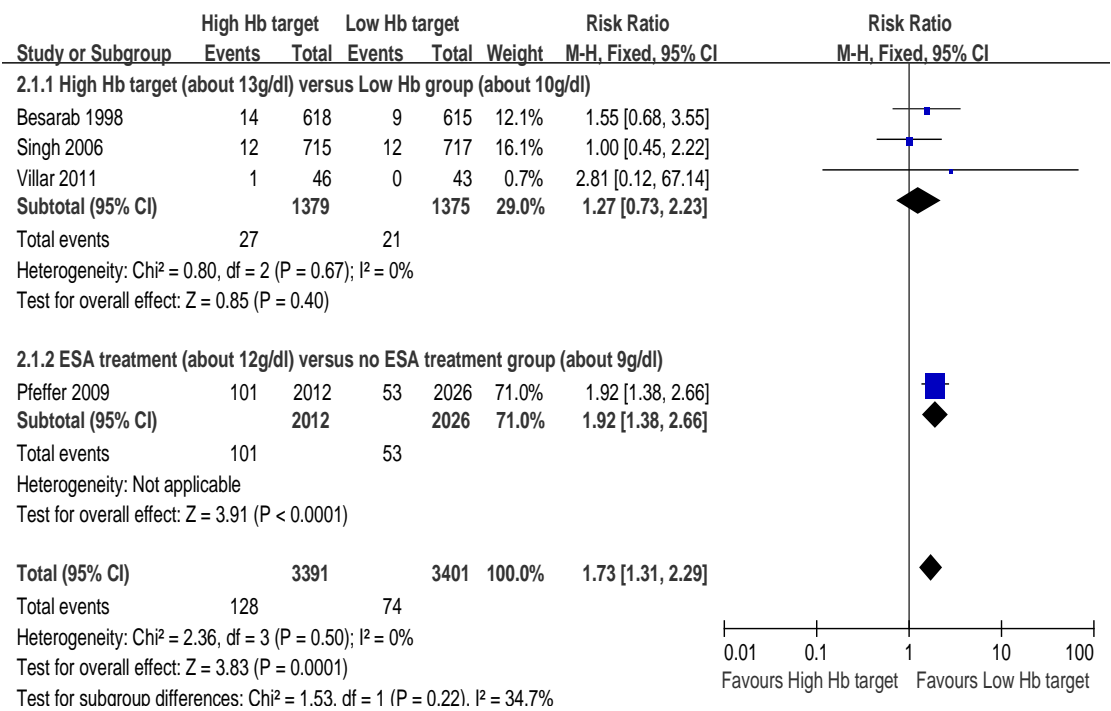

Supplement: Figure S2 — Effect of high versus low Hb targets on stroke in patients with CKD. (PDF) [file pone.0043655.s002.pdf]

**Figure S5. Effect of high versus low Hb targets on hospitalizations in patients with CKD.**

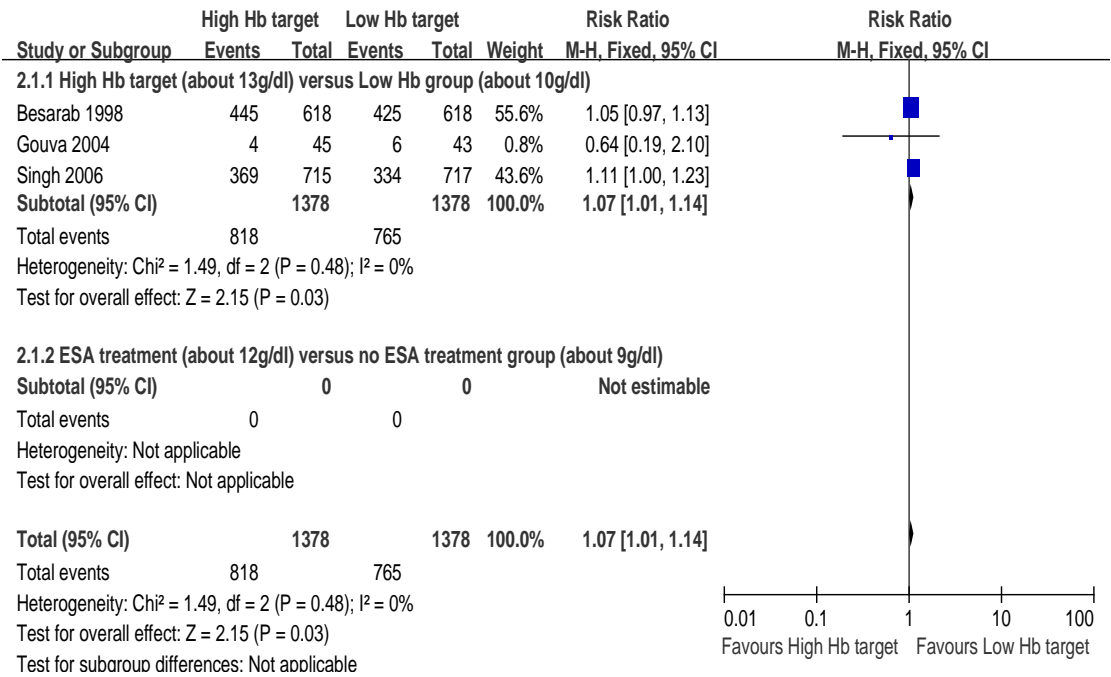

Supplement: Figure S5 — Effect of high versus low Hb targets on hospitalizations in patients with CKD. (PDF) [file pone.0043655.s005.pdf]
