# Supplementary material for: Hemoglobin Targets for Chronic Kidney Disease Patients with Anemia: A Systematic Review and Meta-analysis
Source: PLoS One. 2012 Aug 30;7(8):e43655. doi: 10.1371/journal.pone.0043655 (PMC3431367; doi:10.1371/journal.pone.0043655)

Figure S3 Effect of high versus low Hb targets on non-fatal myocardial infarction in patients with CKD.

Study or Subgroup	High Hb target Events	High Hb target Total	Low Hb target Events	Low Hb target Total	Weight	Risk Ratio M-H, Fixed, 95% CI
**2.1.1 High Hb target (about 13g/dl) versus Low Hb group (about 10g/dl)**						
Besarab 1998	19	618	14	615	25.7%	1.35 [0.68, 2.67]
Druke 2006	14	301	15	302	27.5%	0.94 [0.46, 1.91]
Parfrey 2005	7	296	4	300	7.3%	1.77 [0.52, 6.00]
Ritz 2007	2	88	0	82	0.9%	4.66 [0.23, 95.70]
Singh 2006	18	715	20	717	36.6%	0.90 [0.48, 1.69]
Villar 2011	1	46	1	43	1.9%	0.93 [0.06, 14.48]
**Subtotal (95% CI)**		**2064**		**2059**	**100.0%**	**1.13 [0.79, 1.61]**
Total events	61		54			
Heterogeneity: Chi² = 2.41, df = 5 (P = 0.79); I² = 0%						
Test for overall effect: Z = 0.65 (P = 0.51)						
**2.1.2 ESA treatment (about 12g/dl) versus no ESA treatment group (about 9g/dl)**						
**Subtotal (95% CI)**		**0**		**0**		**Not estimable**
Total events	0		0			
Heterogeneity: Not applicable						
Test for overall effect: Not applicable						
**Total (95% CI)**		**2064**		**2059**	**100.0%**	**1.13 [0.79, 1.61]**
Total events	61		54			
Heterogeneity: Chi² = 2.41, df = 5 (P = 0.79); I² = 0%						
Test for overall effect: Z = 0.65 (P = 0.51)						
Test for subgroup differences: Not applicable						

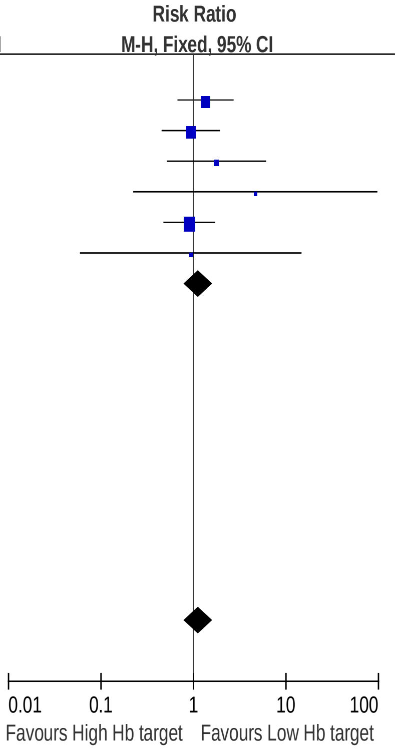

Supplement: Figure S3 — Effect of high versus low Hb targets on non-fatal myocardial infarction in patients with CKD. (PDF) [file pone.0043655.s003.pdf]
