# Supplementary material for: Hemoglobin Targets for Chronic Kidney Disease Patients with Anemia: A Systematic Review and Meta-analysis
Source: PLoS One. 2012 Aug 30;7(8):e43655. doi: 10.1371/journal.pone.0043655 (PMC3431367; doi:10.1371/journal.pone.0043655)

Figure S4. Effect of high versus low Hb targets on RRT in patients with CKD.

S4.1 High Hb versus Low Hb targets (both group treated with ESAs)

	High Hb target		Low Hb target			Risk Ratio
Study or Subgroup	Events	Total	Events	Total	Weight	M-H, Fixed, 95% CI
Akizawa 2011	21	161	26	160	8.0%	0.80 [0.47, 1.37]
Cianciaruso 2008	4	46	4	49	1.2%	1.07 [0.28, 4.01]
Druke 2006	127	301	111	302	34.2%	1.15 [0.94, 1.40]
Gouva 2004	10	45	18	43	5.7%	0.53 [0.28, 1.02]
Levin 2005	11	78	8	74	2.5%	1.30 [0.56, 3.06]
Ritz 2007	2	88	3	82	1.0%	0.62 [0.11, 3.62]
Roger 2004	24	75	15	79	4.5%	1.69 [0.96, 2.96]
Singh 2006	155	715	134	717	41.3%	1.16 [0.94, 1.43]
Villar 2011	2	46	5	43	1.6%	0.37 [0.08, 1.83]
**Total (95% CI)**		**1555**		**1549**	**100.0%**	**1.10 [0.97, 1.25]**
Total events	356		324			

Heterogeneity: Chi² = 11.15, df = 8 (P = 0.19); I² = 28%

Test for overall effect: Z = 1.45 (P = 0.15)

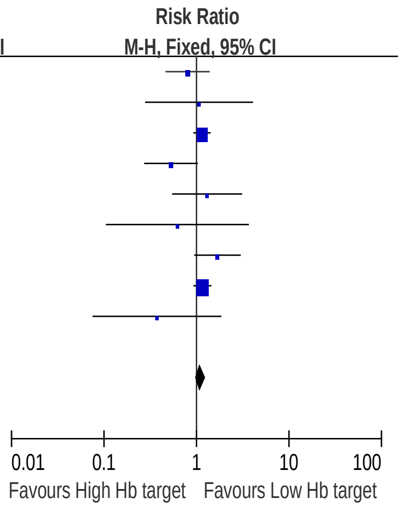

S4.2 ESAs treatment versus no ESAs treatment

	ESA treatment		no ESA treatment			Risk Ratio
Study or Subgroup	Events	Total	Events	Total	Weight	M-H, Random, 95% CI
Kuriyama 1997	14	42	20	31	29.3%	0.52 [0.31, 0.85]
Pfeffer 2009	338	2012	330	2026	45.2%	1.03 [0.90, 1.18]
Revicki 1995	16	43	13	40	25.5%	1.14 [0.63, 2.07]
**Total (95% CI)**		**2097**		**2097**	**100.0%**	**0.86 [0.56, 1.34]**
Total events	368		363			

Heterogeneity: Tau² = 0.11; Chi² = 7.08, df = 2 (P = 0.03); I² = 72%

Test for overall effect: Z = 0.65 (P = 0.52)

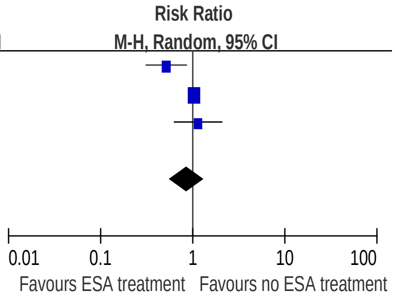

S4.3 Summary of the high Hb versus low Hb group (irrespective of treatment modality)

	High Hb target		Low Hb target			Risk Ratio
Study or Subgroup	Events	Total	Events	Total	Weight	M-H, Random, 95% CI
Akizawa 2011	21	161	26	160	7.1%	0.80 [0.47, 1.37]
Cianciaruso 2008	4	46	4	49	1.5%	1.07 [0.28, 4.01]
Druke 2006	127	301	111	302	19.4%	1.15 [0.94, 1.40]
Gouva 2004	10	45	18	43	5.2%	0.53 [0.28, 1.02]
Kuriyama 1997	14	42	20	31	7.7%	0.52 [0.31, 0.85]
Levin 2005	11	78	8	74	3.3%	1.30 [0.56, 3.06]
Pfeffer 2009	338	2012	330	2026	22.7%	1.03 [0.90, 1.18]
Revicki 1995	16	43	13	40	6.0%	1.14 [0.63, 2.07]
Ritz 2007	2	88	3	82	0.8%	0.62 [0.11, 3.62]
Roger 2004	24	75	15	79	6.5%	1.69 [0.96, 2.96]
Singh 2006	155	715	134	717	18.9%	1.16 [0.94, 1.43]
Villar 2011	2	46	5	43	1.0%	0.37 [0.08, 1.83]
**Total (95% CI)**		**3652**		**3646**	**100.0%**	**1.00 [0.85, 1.18]**
Total events	724		687			

Heterogeneity: Tau² = 0.03; Chi² = 19.65, df = 11 (P = 0.05); I² = 44%

Test for overall effect: Z = 0.00 (P = 1.00)

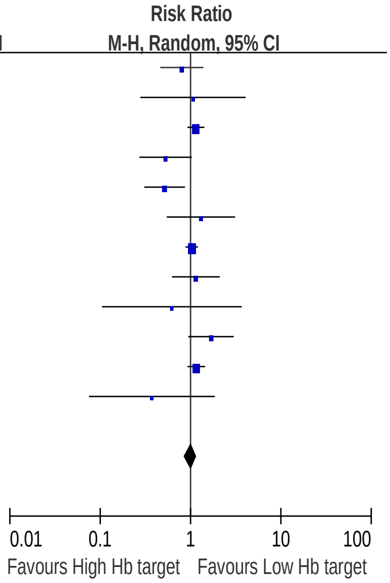

Supplement: Figure S4 — Effect of high versus low Hb targets on RRT in patients with CKD. S4.1. High Hb versus Low Hb targets (both group treated with ESA). S4.2. ESA treatment versus no ESAs treatment. S4.3. Summary of the high Hb versus low Hb group (irrespective of treatment modality). (PDF) [file pone.0043655.s004.pdf]
