# Supplementary material for: Hemoglobin Targets for Chronic Kidney Disease Patients with Anemia: A Systematic Review and Meta-analysis
Source: PLoS One. 2012 Aug 30;7(8):e43655. doi: 10.1371/journal.pone.0043655 (PMC3431367; doi:10.1371/journal.pone.0043655)
Supplement: Table S1 — Search Strategy. (PDF) [file pone.0043655.s006.pdf]

Table S1. Search Strategy

Medline

#1.Kidney Diseases/exp

#2.Kidney Failure, Chronic/exp

#3.Renal Insufficiency/exp

#4.Uremia/exp

#5.Renal Dialysis/exp

#6.Dialysis/exp

#7.Peritoneal Dialysis/exp

#8.Hemodialysis, Home/exp

#9.Hematinics/exp

#10.Anemia/exp

#11.(#1 or #2 or #3 or #4 or #5 or #6 or #7 or #8)

#12.(#9 and #10 and #11)

Embase

#1. Chronic kidney disease/exp

#2. Kidney failure/exp

#3.Chronic kidney failure/exp

#4.kidney disease/exp

#5.Uremia/exp

#6.Dialysis/exp

#7.Continuous ambulatory peritoneal dialysis/exp

#8.hemodialysis/exp

#9.Renal replacement therapy/exp

#10.Peritoneal dialysis/exp

#11.Equilibrium dialysis/exp

#12.Extended daily dialysis/exp

#13.anemia/exp

#14.Erythropoiesis stimulating and agents/exp

#15.(#1 or #2 or #3 or #4 or #5 or #6 or #7 or #8 or #9 or #10 or #11 or #12)

#16.(#13 and #14 and #15)

The Cochrane Database of Systematic Reviews

#1.Renal Insufficiency, Chronic/exp

#2.Renal Replacement Therapy/exp

#3.Renal Insufficiency/exp

#4.Kidney Failure, Chronic/exp

#5.Renal Dialysis/exp

#6.Hemodialysis, Home/exp

#7.Uremia/exp

#8.Peritoneal Dialysis, Continuous Ambulatory/exp

#9.Dialysis/exp

#10.Peritoneal Dialysis/exp

#11.Anemia/exp

#12.Hematinics/exp

#13.(#1 or #2 or #3 or #4 or #5 or #6 or #7 or #8 or #9 or #10)

#14.(#12 and #13)
