# Supplementary material for: Hemoglobin Targets for Chronic Kidney Disease Patients with Anemia: A Systematic Review and Meta-analysis
Source: PLoS One. 2012 Aug 30;7(8):e43655. doi: 10.1371/journal.pone.0043655 (PMC3431367; doi:10.1371/journal.pone.0043655)
Supplement: Table S2 — Characteristics of studies included in our meta-analysis. (PDF) [file pone.0043655.s007.pdf]

Table S2. Characteristics of studies included in our meta-analysis.

Study	Treatment Modality	Randomized Intervention	Other Intervention
**High Hb levels versus Low Hb levels**			
Singh et al.[4]	predialysis	ESAs versus ESAs	None indicated
Drueke et al.[5]	predialysis	ESAs versus ESAs	IV or oral iron (dose NA)
Akizawa et al.[14]	predialysis	ESAs versus ESAs	Iron (dosage/route NA) to maintain transferrin saturation >20% or serum ferritin >100ng/ml
Villar et al.[15]	predialysis	ESAs versus ESAs	Iron (route NA) dosage was 69.7±91.4mg/d for low Hb group; 91.6±100.3mg/d for high Hb
Cianciaruso et al. [16]	predialysis	ESAs versus ESAs	Oral or IV Fe (transferrin saturation >20% and serum ferritin >60ng/L)
Ritz et al.[17]	predialysis	ESAs versus ESAs	None indicated
Parfrey et al.[18]	hemodialysis	ESAs versus ESAs	None indicated
Levin et al.[19]	predialysis	ESAs versus ESAs	Oral or IV Fe (target transferrin saturation >20%; serum ferritin >60ng/L)
Roger et al.[20]	predialysis	ESAs versus ESAs	Oral or IV Fe (transferrin saturation >20% and serum ferritin >100ug/L)
Gouva et al.[21]	predialysis	ESAs versus ESAs	Oral or IV Fe (dose/route NA) to patients with transferrin saturation <20%
Furuland et al.[22]	mixed	ESAs versus ESAs	Oral or IV Fe (target transferrin saturation >20%)
Foley et al.[23]	hemodialysis	ESAs versus ESAs	None indicated
Conlon et al.[24]	hemodialysis	ESAs versus ESAs	IV iron (dose NA)
Berns et al.[25]	hemodialysis	ESAs versus ESAs	None indicated
Besarab et al.[26]	hemodialysis	ESAs versus ESAs	Iron at investigators'discretion
CESG(a substudy) [27]	hemodialysis	EPO versus EPO	Oral or IV Fe (dose NA) to patients with a serum ferritin <250ug/L
**ESA versus no ESA treatment**			
Patel et al.[28]	predialysis	EPO versus Control	None indicated
Preffer et al.[29]	predialysis	ESAs versus Placeo	None indicated
Pappas et al.[30]	predialysis	EPO versus no treatment	Oral iron (100mg/d)to maintain transferrin saturation>20%; serum ferritin>150ug/L) for the rhuEPO treatment groupNA for the control group
Kuriyama et al.[31]	predialysis	EPO versus no treatment	Iron at investigators'discretion
Revicki et al.[32]	predialysis	EPO versus no treatment	Iron < 200mg/d (route NA) if serum iron below 50ug/dl
Nissenson et al.[33]	peritoneal dialysis	EPO versus Placeo	Fe (dose/route NA)
Sikole et al.[34]	hemodialysis	EPO versus Control	None indicated
Teehan et al.[35]	predialysis	EPO versus Placeo	None indicated
CESG [27]	hemodialysis	EPO versus Placeo	Oral or IV Fe (dose NA) to patients with a serum ferritin <250ug/L

NA, not available; Control, without ESAs treatment.
