# Supplementary material for: Hemoglobin Targets for Chronic Kidney Disease Patients with Anemia: A Systematic Review and Meta-analysis
Source: PLoS One. 2012 Aug 30;7(8):e43655. doi: 10.1371/journal.pone.0043655 (PMC3431367; doi:10.1371/journal.pone.0043655)
Supplement: Table S3 — Summary of Hb levels in our included studies. (PDF) [file pone.0043655.s008.pdf]

Table S3. Summary of Hb levels in our included studies.

		High Hb Group				Low Hb Group			
Study	Follow-up (months)	N	Targeted Hb Levels (g/dL)	Basic Hb Levels (g/dL)	Achieved Hb Levels (g/dL)	N	Targeted Hb Levels (g/dL)	Basic Hb Levels (g/dL)	Achieved Hb Levels (g/dL)
**High Hb levels versus Low Hb levels**									
Singh et al.[4]	16	715	13.0-15.0	10.1±0.9	13.5	717	10.5-11.5	10.1±0.9	11.3
Drueke et al.[5]	36	301	13.0-15.0	11.6±0.6	13.4	302	10.5-11.5	11.6±0.6	11.6
Akizawa et al.[14]	12	160	11.0-13.0	9.15±0.79	11.0-13.0	160	9.0-11.0	9.18±0.86	9.0-11.0
Villar et al.[15]	24	46	13-14.9	114.2±10.6	>13.0	43	11-12.9	114.7±8.10	<12.0
Cianciaruso et al.[16]	12	46	12.0-14.0	11.6±0.7	12.4±1.1	49	9.0-10.5	11.7±0.8	11.3±1.3
Ritz et al.[17]	15	88	13-15	11.3-12.2	13.5	82	10.5-11.5	11.3-12.0	12.1
Parfrey et al.[18]	18.5	296	13.5-14.5	11.0±1.2	13.5±1.5	300	9.5-11.5	11.0±1.2	10.9±1.2
Levin et al.[19]	24	78	12.0-14.0	11.7±0.76	12.5±0.87	74	9.0-10.5	9.0±0.5	9.0±0.5
Roger et al.[20]	24	75	12.0-13.0	11.0-13.0	11.5-12.9	79	9.0-10.0	11.0-13.0	10.0-11.0
Gouva et al.[21]	22.5	45	11.0-11.7	10.1±0.5	12.9±0.4	43	NA[b]	10.1±0.6	10.3±1.0
Furuland et al.[22]	12	216	13.5-16.0	10.9±1.1	13.5-16.0	200	9.0-12.0	11.0±0.9	11.30±1.3
Foley et al.[23]	12	73	13.0-14.0	12.2	13.0-14.0	73	9.5-10.5	10.4	9.5-10.5
Conlon et al.[24]	7.5	15	42±3(%)[a]	29.1±2.4(%)[a]	48±5.2(%)[a]	16	30±3(%)[a]	29.1±2.1(%)[a]	30±3(%)[a]
Berns et al.[25]	12	14	42±3(%)[a]	30.7±0.7(%)[a]	42.0±1.1(%)[a]	14	NA[b]	30.6±0.7(%)[a]	30.4±1.1(%)[a]
Besarab et al.[26]	36	618	14.0±1.0	10.2	12.7-13.3	615	10.0±1.0	10.2	10.0±1.0
CESG(a substudy).[27]	6	38	11.5-13	7.1±1.2	11.7±1.4	40	9.5-11	6.9±1.0	10.2±1
**ESA versus no ESA treatment**									
Patel et al.[28]	4.75	118	NA[b]	10.1±0.8	11	39	NA[b]	10.2±0.9	10.5
Pfeffer et al.[29]	29.1	2012	12.0-12.8	9.7-11.0	12.0-12.8	2026	9.9-11.3	9.8-10.9	9.9-11.3
Pappas et al.[30]	12	15	13	10.1±0.6	13.6±0.1	16	NA[b]	10.4±0.5	10.2±1.2
Kuriyama et al.[31]	9	42	11.0-11.7	9	11.8±1.3	31	NA[b]	9	8.4±0.6
Revicki et al.[32]	12	43	11.6-12.0	8.9	12	40	NA[b]	8.9	8.9±1.2
Nissenson et al.[33]	6-9	78	10.7-12.7	8	11.2	74	10-11.7	8	8
Sikole et al.[34]	12	19	10-11.7	6.7	11.3	19	NA[b]	6.7	8.3
Teehan et al.[35]	6	86	NA[b]	NA[b]	12.8-13.3	31	12.3-13.3	NA[b]	10
CESG.[27]	6	38	11.5-13	7.1±1.2	11.7±1.4	40	NA[b]	7.1±0.9	7.4±1.2

a. Hematocrit value.

b. NA, not available.
