# Supplementary material for: Hemoglobin Targets for Chronic Kidney Disease Patients with Anemia: A Systematic Review and Meta-analysis
Source: PLoS One. 2012 Aug 30;7(8):e43655. doi: 10.1371/journal.pone.0043655 (PMC3431367; doi:10.1371/journal.pone.0043655)
Supplement: Table S4 — The quality of evidence and the strengths of recommendations. (PDF) [file pone.0043655.s009.pdf]

Table S4. The quality of evidence and the strengths of recommendations.

Outcomes	Illustrative comparative risks* (95% CI)		Relative effect (95% CI)	No of Participants (studies)	Quality of the evidence (GRADE)
	Assumed risk	Corresponding risk			
	Control				
Mortality	Study population		RR 1.10<br>(1.00 to 1.21)	9859<br>(18 studies)	⊕⊕⊕◯<br>moderate
	138 per 1000	152 per 1000<br>(138 to 167)			
	Moderate				
Hypertension	Study population		RR 1.40<br>(1.12 to 1.75)	6755<br>(8 studies)	⊕⊕⊕◯<br>moderate
	61 per 1000	86 per 1000<br>(69 to 107)			
	Moderate				
Stroke	Study population		RR 1.73<br>(1.31 to 2.29)	6792<br>(4 studies)	⊕⊕⊕⊕<br>high
	22 per 1000	39 per 1000<br>(29 to 51)			
	Moderate				
Non-fatal myocardial infarction	Study population		RR 1.13<br>(0.79 to 1.62)	4122<br>(6 studies)	⊕⊕⊕◯<br>moderate
	26 per 1000	30 per 1000<br>(21 to 42)			
	Moderate				
Renal Replacement Therapy	Study population		RR 1.00<br>(0.85 to 1.18)	7298<br>(12 studies)	⊕⊕⊕◯<br>moderate
	89 per 1000	89 per 1000<br>(76 to 105)			
	Moderate				
Hospitalizations	Study population		RR 1.07<br>(1.00 to 1.14)	2753<br>(3 studies)	⊕⊕⊕◯<br>moderate
	556 per 1000	595 per 1000<br>(556 to 634)			
	Moderate				
